# Host-Dependent Modifications of Packaged Alphavirus Genomic RNA Influence Virus Replication in Mammalian Cells

**DOI:** 10.3390/v14122606

**Published:** 2022-11-23

**Authors:** John M. Crawford, Liewei L. Yan, Hani Zaher, Richard W. Hardy

**Affiliations:** 1 Department of Biology, Indiana University, Bloomington, IN 47405, USA; 2 Department of Biology, Washington University, St. Louis, MO 63130, USA

**Keywords:** virus infectivity, host dependence, RNA modifications

## Abstract

Alphaviruses must interact efficiently with two distinct host environments in order to replicate and transmit between vertebrate and mosquito hosts. Some host-origin-dependent differences in virus particle composition that appear to facilitate the transmission cycle are known. However, the impact of host-mediated modification of packaged viral genomic RNA on subsequent infection has not been previously investigated. Here we show that in human (HEK-293) cells, mosquito-derived Sindbis virus (SINV) replicates and spreads faster, producing a more infectious virus than its mammalian-derived counterpart. This enhanced replication is neither a result of differences in the stability nor the production of the infecting genomic RNA. Nevertheless, purified genomic RNA from mosquito-derived SINV established infection in HEK-293 cells more efficiently than that of mammalian-derived SINV, indicating that the genomic RNA itself is different between the two producing hosts and this difference is a determinant of infection. In agreement with this idea, we show that mosquito-derived SINV genomic RNA is a more active template for translation than mammalian-derived SINV genomic RNA, and we attribute this difference to host-dependent changes in modification of packaged genomic RNA as determined by LC/MS-MS. Our data support the hypothesis that among other factors, the host-dependent modification profile of the packaged vRNA is likely to play an important role in the efficiency of SINV infection and replication in mammalian cells.

## 1. Introduction

Arthropod borne viruses (arboviruses) are characterized by their ability to replicate in both vertebrate and invertebrate hosts. These viruses use the same, limited set of genes to replicate and transmit between the vertebrate host and mosquito vector. Arboviruses are adept at interacting with two distinct hosts, programming efficient replication in both host environments, and priming the progeny for spread to a distinct host environment. There is currently a limited understanding of the molecular determinants of transmission that facilitate efficient alternate infection of host species.

Alphaviruses are arboviruses and are predominantly vectored between vertebrates by mosquitoes. Alphaviruses are divided into two main groups based on the disease they cause. The first group is the arthritogenic viruses, such as Sindbis virus (SINV) and Chikungunya virus (CHIKV), which cause debilitating but non-life-threatening polyarthritis. The second group is the encephalitogenic viruses, such as Venezuelan and Eastern equine encephalitis virus, VEEV/EEEV, which can cause fatal encephalitis. There are currently no effective vaccines for alphaviruses, and outbreaks—particularly of CHIKV—occur regularly around the world. It is therefore important to improve our understanding of alphavirus replication and interaction with both vertebrate and insect hosts. In the work described here, we utilize SINV, the type species of the Alphavirus genus. SINV is an enveloped, single-stranded, positive-sense RNA virus with an approximately 11.7 kb genome. In vertebrate cells, SINV causes an acute, cytolytic infection, whereas in arthropod cells the infection is persistent and noncytolytic.

There is good evidence of alphaviruses’ capabilities to prime the progeny produced in arthropod cells for infection in vertebrate cells, as it has been shown that arthropod-derived alphaviruses have increased infectivity on vertebrate cells, and vertebrate-derived viruses initiate infection of mosquito midgut more efficiently [1,2]. Similarly, it has been shown that alphaviruses acquire host-specific alterations that improve their ability to establish infection in the new host; these host-specific differences range from differential packaging of host components such as 18S rRNAs that improve the viral infectivity on mammalian cells to glycosylation of the envelope glycoproteins, which allows for targeted infection of certain mammalian cell types [2,3,4,5,6]. Moreover, though a newer field of study, there is evidence of specific RNA modification of viral RNA in the host having various effects on viral RNA function. RNA methylation is driven by host methyltransferase genes, such as METTL3 or TRDMT1. Methyltransferases transfer a methyl group from a methyl carrier molecule, such as S-adenosyl methionine, to certain nucleotides. These methylated nucleotides are recognized by methyl readers that confer different effects on the RNA. Indeed, RNA methylation has been documented to modulate a range of steps in RNA metabolism, including—but not limited to—mRNA stability, translation, localization, and for viral RNA, viral replication [7,8,9,10,11,12,13,14,15,16,17]. Recent studies have focused heavily on the role of m6A modifications of viral RNA and how those modifications affect viral replication [18,19]. For instance, it has been shown that N6-methyladenosine (m6A) post-transcriptionally regulates the RNA function of the flavivirus hepatitis C virus (HCV) in a negative manner in mammalian cells [14]. There is also evidence of the hepatitis B virus’ (HBV) life cycle being regulated by m6A modification of the pregenomic RNA intermediate wherein the m6A modifications are required for efficient reverse transcription as well as stabilizing HBV transcripts and ultimately affecting protein expression [13,20]. In previous work we showed that SINV acquires 5-methylcytosince (m5C) in mosquito cells [15]. This m5C modification of SINV RNA was shown to have a proviral effect on the progeny virus used to infect mammalian cells, as ectopic expression of the m5C writer in mosquito cells resulted in a higher specific infectivity as compared to the control [15]. Further indirect evidence of utilization of host methyltransferases by viruses is provided in multiple reports, showing that virus infection leads to increased expression of methyltransferase genes [9,15]. Thus, it is important to explore the role that RNA modification may play in alphavirus infectivity and whether the modification profiles of viral RNA differ between viruses derived from arthropod and mammalian hosts.

Here, we show that in human (HEK-293) cells, mosquito-derived SINV replicates faster and produces more infectious viruses. Mosquito-derived SINV also has increased translation compared to mammalian-derived SINV, and this increased translation is not a result of either incoming genomic RNA stability or increased vRNA levels in the cell. We also show that purified genomic vRNA phenocopies infectivity of purified viruses, and we attribute these differences to host-dependent changes in modification of packaged genomic RNA as determined by LC/MS-MS. Our data support the hypothesis that among other factors [2,3,4], the host-dependent modification profile of the packaged vRNA plays an important role in the efficiency of SINV infection and replication in mammalian cells.

## 2. Materials and Methods

### 2.1. Insect and Mammalian Cell Culture

C7/10 Aedes albopictus cells were grown at 28 °C under 5% ambient CO_2_ in 1× Minimal Essential Media (Corning) supplemented with 10% heat-inactivated fetal bovine serum (Corning), 1% L-glutamine (Corning), 1% non-essential amino acids (Corning), and 1% antibiotic–antimycotic solution (Corning). Vertebrate baby hamster kidney fibroblast or BHK-21 cells, as well as human embryonic kidney epithelial or HEK293 cells, were grown at 37 °C under 5% ambient CO_2_ in the same 1× Minimal Essential Media (Corning) as the C7/10 cells.

### 2.2. Obtaining Viruses

BHK-21-derived viruses were obtained by in vitro transcription of SINV full-length plasmid using SP6 RNA polymerase (NEB). IVTs were then transfected into confluent BHK cells using LTX transfection mix (Invitrogen), using manufacturer’s protocol, in a T25 flask (CellStar) with serum-free virus production media (Gibco). Four hours post-transfection, the SF-VPM was removed and replaced with fresh 1× MEM supplemented with 10% FBS. Twenty-four hours post-transfection, the viral supernatant was harvested and purified by centrifugation at 43,000× *g* for 2.5 h over a 27% *w/v* sucrose cushion in HNE buffer (150 mM NaCL, 20 mM HEPES, 0.1 mM EDTA). The media was discarded, and the viral pellet was resuspended in 500 µL of HNE buffer. Plaque assays on BHK-21 cells were then performed to determine viral titer.

C7/10-derived virus was obtained by infecting confluent C7/10 cells in a 150 mm dish (CellStar) with P0 BHK-derived virus at an MOI of 0.1 PFU/cell. The inoculum was left on for 3 h, after which the media were replaced, cells were washed with PBS, and fresh 1× MEM was added. Forty-eight hours postinfection, the viral supernatant was harvested, purified and plaque-assayed as previously described.

### 2.3. Live-Cell Imaging and Single-Step Growth Curve

Live-cell imaging was performed using IncuCyte live-cell analyses system (Essen Biosciences, Ann Arbor, MI, USA). HEK293 cells were plated in a 24-well plate (CellStar) at 70–80% confluency and synchronously infected with Sindbis virus with a translationally fused green fluorescent protein in the nonstructural protein 3 (SINV-nsP3-GFP) at an MOI of 5 PFU per cell. Viral inoculum was allowed to incubate on the cells for 30 min at 4 °C before being replaced with prewarmed media. Bright light and fluorescent microscopy images were taken every 2 h for 30 h. Mean fluorescent count was graphed with standard error mean calculated from 3 replicate wells in the plate with 9 images taken within each well. At the time points indicated, total viral supernatant was removed from the wells and replaced with fresh 1× MEM. Viral supernatant from each time point was then used for plaque assays on BHK-21 cells.

### 2.4. Virion RNA Extractions, Transfections, and Luciferase Assays

BHK-21 and C7/10 cells were infected with SINV-nLuc, and viral supernatant was obtained at 24 and 48 hpi, respectively. Viral supernatant was purified over a 27% *w/v* sucrose cushion in HNE buffer for 2.5 h at 43,000× *g* before being resuspended in HNE buffer. Virion-encapsidated RNA was extracted from the BHK-21- and C7/10-derived viruses (SINV-nsP3-NLuc) using TRiZOL (Sigma Aldrich) following the manufacturer’s protocol. Postextraction, RNAs were DNase (RQ1 RNase-free DNase, NEB)-treated using the manufacturer’s protocol to remove cellular contaminants, and viral RNA copies were quantified via quantitative RT-PCR using primers probing for SINV E1 genomic region and a standard curve comprised of linearized SINV infectious clone containing the full-length viral genome. To determine replication kinetics of Sindbis virion RNA derived from BHK-21 or C7/10 cells, HEK-293 cells were transfected or infected with equal copies of virion-isolated RNA or purified virions (10^8^ copies, quantified using qRT-PCR) in serum-free Opti-MEM (Gibco) using LTX transfection mix (Invitrogen). Transfection was carried out for 4 h before the transfection inoculum was removed and replaced with 1× MEM. Infections were carried out synchronously for 30 min at 4 °C before prewarmed 1× MEM was added. Cells were harvested at indicated times post-transfection and homogenized in 1× Cell Culture Lysis Reagent (Promega). Samples were mixed with NanoGlo luciferase Reagent (Promega), incubated at room temperature for 5 min, then recorded for luminescence using a Synergy H1 microplate reader (BioTech instruments). Total cellular RNA was obtained using the BioRad Aurum total RNA mini kit according to the manufacturer’s protocol. Genomic vRNA copies were then quantified via quantitative RT-PCR using primers probing for SINV nsP1 genomic region to detect only (+) vRNA in the cell and a standard curve comprised of linearized SINV infectious clone containing the full-length viral genome.

### 2.5. Input RNA Decay

Input RNA Decay assay was adapted from a previously described protocol [21,22]. HEK-293 cells were seeded at 80% confluency in a 24-well plate, and 30 min prior to infection, 3× well volume of 1× MEM supplemented with 4SU (Sigma-Aldrich) at a concentration of 50 µM was added to each well. At the time of infection, the 1× MEM supplemented with 4SU was removed and 250 uL of inoculum was added to infect the cells at an MOI of 10 PFU/cell with either BHK-21 derived, or C7/10 derived SINV. The 24-well plate was then placed at 4 °C for 30 min for a synchronous infection of virus. After 30 min, the plate was removed from 4 °C, the inoculum was removed, and prewarmed 1× MEM supplemented with 50 µM 4SU was added to the cells to allow virus to be absorbed. Five minutes later, the media were removed, the cells were washed 3× with PBS to remove any unbound virus, 1× MEM supplemented with 50 µM 4SU was added, and the 0 min postinfection time point was harvested. Time points were harvested at 0, 45, 90, and 135 min postinfection. Media were removed and RNA Lysis Solution was added to each well before being moved to a clean 1.5 mL tube for further RNA extraction using the BioRad Aurum Total RNA mini kit according to the manufacturer’s protocol. Streptavidin agarose beads (ThermoFisher) were incubated with EZ-Link HPDP-Biotin (ThermoFisher) at 10× the binding capacity for 30 min at 25 °C under gentle agitation. After incubation, the biotin-bound beads were washed 3× with PBS before being added to 1 µg of total cellular RNA for each sample. The biotin-bound beads and RNA were incubated for 1.5 h at 20 °C under gentile agitation. Following incubation, the beads were spun down at 6000× *g* for 5 min and the unbound, unlabeled RNA supernatant was removed. The unbound RNA was then cleaned up using the RNeasy mini kit (Qiagen) following the manufacturer’s protocol and quantified by Nanodrop to determine RNA concentration. The purified, unlabeled RNA was then used for qRT-PCR to determine relative unlabeled viral RNA at each time point compared to the 0 min postinfection time point. The purified, unlabeled, cellular RNA was then used as a template to synthesize cDNA using M-MuLV Reverse Transcriptase (NEB) with random hexamer primers (Integrated DNA Technologies). Quantitative RT-PCR analysis was performed using Brilliant III SYBR green QPCR master mix (Thomas Scientific) with SINV E1 genomic region-specific primers and Human 18S primers according to the manufacturer’s protocol and with the Applied Bioscience StepOnePlus qRT-PCR machine (Life Technologies). The relative abundance of SINV genomic RNA was determined by normalizing to Human 18S expression using the delta-delta comparative threshold method (ΔΔCT) and compared back to the 0 min postinfection sample.

### 2.6. Western Blotting

HEK-293 were infected with 10^8^ genomes of purified SINV-nsP3-NLuc derived from either C7/10 or BHK-21 cells, as described previously. At 9 h postinfection, cells were lysed and harvested using Lysis Buffer II (10 mM Tris pH 7.4, 150 mM NaCl, 1% Triton-X100, 10 mM NaH_2_PO_4_, 5 mM EDTA, and 1× PMSF). Protein was extracted by vortexing for 10 s every 2 min over 10 min. At the end of 10 min, the samples were vortexed one last time then spun down for 10 min at 10,000 RPM. The supernatant was extracted and moved to a fresh tube. The samples were then mixed with SDS-loading buffer to a concentration of 1× and heated at 95 °C for 5 min. The samples were then run through a 10% SDS-PAGE gel and transferred to a PVDF membrane. The membrane was blocked in 5% TBSM (Tris Buffer Saline 1× + 5% Dry Milk) for 1 h at room temperature. Following blocking, the membrane was incubated with rabbit anti-nsP2 or rabbit anti-β actin at 4 °C overnight. Following incubation with primary antibody, the membrane was washed with TBST prior to incubation with goat anti-rabbit AlexaFluor 750 (ThermoFisher) for 1 h at room temperature. Following secondary antibody incubation, the membrane was imaged using a BioRad ChemiDoc MP Imaging System. Band intensity was determined using ImageStudio Lite Ver. 5.2. Band intensity is represented as a ratio of nsP2 signal to β-actin signal and standardized to the BHK-21-derived virus sample.

### 2.7. Quantification of RNA Modifications by LC-MS/MS

BHK-21- and C7/10-derived Sindbis virus were produced as explained in Obtaining Viruses. Virion-encapsidated RNA was harvested using TRiZOL (Sigma Aldrich) following the manufacturer’s protocol. Total RNA (~1 µg) was digested by nuclease P1 (10 Units) at 50 °C for 16 h. Additional Tris pH 7.5 was then added to a final concentration of 100 mM to adjust the pH, which was followed by the addition of calf intestinal alkaline phosphatase (CIP, NEB, 2 Units). The mixture was incubated at 37 °C for 1 h to convert nucleotide 5′-monophosophates to their respective nucleosides. A total of 10µL of RNA samples were diluted to 30µL and filtered (0.22 µm pore size). A total of 10 µL of the sample was used for LC-MS/MS. Briefly, nucleosides were separated on a C18 column (Zorbax Eclipse Plus C18 column, 2.1 × 50 mm, 1.8 micron) paired with an Agilent 6490 QQQ Triple-quadrupole LC mass spectrometer using multiple-reaction monitoring in positive-ion mode. The nucleosides were quantified using the retention time of the pure standards and the nucleoside-to-base-ion mass transitions of 268.1 to 136 (A), 244.1 to 113 (C), 284.2 to 152 (G), 300 to 168.1 (8-oxoG), 282.2 to 150 (m1A), 298 to 166 (m1G), 258 to 126 (m3C and m5C), 282.1 to 150 (m6A), 298 to 166 (m7G). Standard calibration curves were generated for each nucleoside by fitting the signal intensities against concentrations of pure nucleoside preparations. The curves were used to determine the concentration of the respective nucleoside in the sample. The A, G, and C standards were purchased from ACROS ORGANICS; m5C was purchased from BioVision; m7G, m1G, and m3C were purchased from Carbosynth; m6G and m6A were purchased from Berry’s Associates; and m1A was purchased from Cayman Chemical Company. The modification level on the nucleosides was calculated as the ratio of modified: unmodified.

### 2.8. In Vitro Translation

BHK-21 and C7/10 cells were infected with SINV-nLuc, and viral supernatant was obtained at 24 and 48 hpi, respectively. Viral supernatant was purified over a 27% *w/v* sucrose cushion in HNE buffer for 2.5 h at 43,000× *g* before being resuspended in HNE buffer. Virion-encapsidated RNA was extracted from the BHK-21- and C7/10-derived viruses (SINV-nsP3-NLuc) using TRiZOL (Sigma Aldrich) following the manufacturer’s protocol. Postextraction, RNAs were DNase (RQ1 RNase-free DNase, NEB)-treated using the manufacturer’s protocol to remove cellular contaminants, and viral RNA copies were quantified via quantitative RT-PCR using primers probing for SINV E1 genomic region and a standard curve comprised of linearized SINV infectious clone containing the full-length viral genome. Rabbit Reticulocyte Lysate, Nuclease-Treated (Promega), was used according to the manufacturer’s protocol. Briefly, 10^8^ genomes of BHK-21- and C7/10-derived purified vRNA was added to the Rabbit Reticulocyte Lysate and incubated at 30 °C for 90 min. A plasmid containing the full-length genome of SINV-nsP3-NLuc was used as a template for in vitro transcription using MEGAscript SP6 Transcription Kit (ThermoFisher) following the manufacturer’s protocol. As a control, 2 µG of the in vitro transcribed RNA was added to the Rabbit Reticulocyte Lysate following the manufacturer’s protocol. Following incubation, 5 µL of the in vitro translation was added to 25 µL of NanoGlo luciferase Reagent (Promega), incubated at room temperature for 5 min then recorded for luminescence using a Synergy H1 microplate reader (BioTech instruments).

### 2.9. Statistical Analysis of Experimental Data

All statistical analyses were performed using GraphPad Prism 9 (GraphPad Software Inc., San Diego, CA, USA).

### 2.10. Graphics

Graphical assets made in ©BioRender—biorender.com. Accessed on 25 August 2022.

## 3. Results

### 3.1. Mosquito-Derived Virus Replicates Faster in HEK-293 Cells

**Figure 1:**
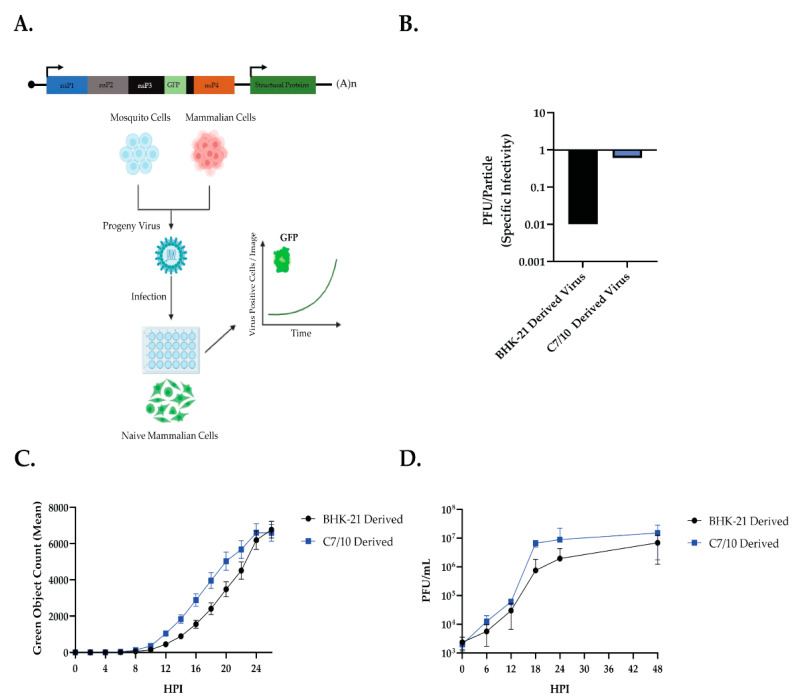
Production of virus and spread in HEK293 cells. (**A**) Schematic representation of the experiment. SINV expressing green fluorescent protein from an nsP3 fusion, SINV-nsP3-GFP, was grown in BHK-21 cells and C7/10 cells. The progeny viruses were then purified over a sucrose cushion, as explained in Materials and Methods, and used to infect HEK-293 cells synchronously at an MOI of 5 PFU/cell. Virus growth in cells, plated on a 24-well plate, was measured in real time by imaging and quantifying the number of green cells (Green Object Count/Image) expressing the virus-encoded GFP protein over a period of 24 h, using live cell imaging. Supernatant samples were taken at the indicated time points (**D**) in a single-step growth curve for tittering on BHK-21 cells. (**B**) Specific infectivity (PFU/particle) of SINV-nsP3-GFP virus used for the experiment. (**C**) Live-cell imaging of HEK-293 cells infected with GFP-tagged SINV derived from BHK-21 cells (black) or C7/10 cells (blue) at an MOI of 5PFU/cell. Error bars represent SEM of biological replicates (*n* = 3) Two-way ANOVA with Sidak’s multiple comparisons: time—*p* < 0.0001, BHK/C7-derived virus—*p* < 0.0001, time × BHK/C7-derived virus—*p* = 0.0057. (**D**) Single-step growth curve of SINV-nsP3-GFP derived from BHK-21 cells (black) or C7/10 cells (blue) on HEK-293 cells, plaqued on BHK-21 cells. Error bars represent SEM of biological replicates (*n* = 3).

The growth of the Sindbis virus (SINV) derived from both mosquito and mammalian cells on human embryonic kidney 293 (HEK-293) cells was examined (Figure 1A). SINV with a translationally fused green fluorescent protein (GFP) reporter in nonstructural protein 3 (SINV-nsP3-GFP) [23] was grown in either baby hamster kidney (BHK-21) cells or Aedes albopictus (C7/10) cells for 24 or 48 h, respectively. The virus containing supernatant was then removed and purified over a 27% sucrose cushion in HNE buffer before being resuspended in HNE buffer. The BHK-21- and C7/10-derived viruses were plaqued and used for reverse transcription to determine total genomes using qRT-PCR. The C7/10-derived virus had a specific infectivity (PFU/Genomes) of 0.61 and the BHK-21-derived virus had a specific infectivity of 0.01 (Figure 1B). These numbers fall within the previously reported range for Sindbis virus-specific infectivity [1,3,24]. HEK-293s were synchronously infected at an MOI of 5 PFU/cell with either mammalian-derived (BHK-21 derived) or mosquito-derived (C7/10 derived) SINV-nsP3-GFP (Figure 1C,D). After absorption at 4 °C the cells were removed, and prewarmed (37 °C) media was added. The HEK-293 cells were analyzed by IncuCyte, measuring fluorescence per cell, and growth medium was sampled at each time point to determine infectious titer (Figure 1D). The mosquito-derived virus not only replicated faster but also produced a more infectious virus during infection (Figure 1C,D). Two-way ANOVA with Sidak’s multiple comparison test showed an effect on both time and viral progeny background from the Incucyte data: time—*p* < 0.0001, viral progeny background—*p* < 0.0001, time × viral progeny background—*p* = 0.0057 (Figure 1C). From this, we conclude that mosquito-derived viruses are replicating faster than mammalian-derived viruses on HEK-293 cells, as well as producing more infectious viruses. These results are in line with previous data suggesting that mosquito-derived SINV is more infectious on mammalian cells [1,3,5].

### 3.2. Mosquito-Derived Virus has Increased Translational Activity

**Figure 2:**
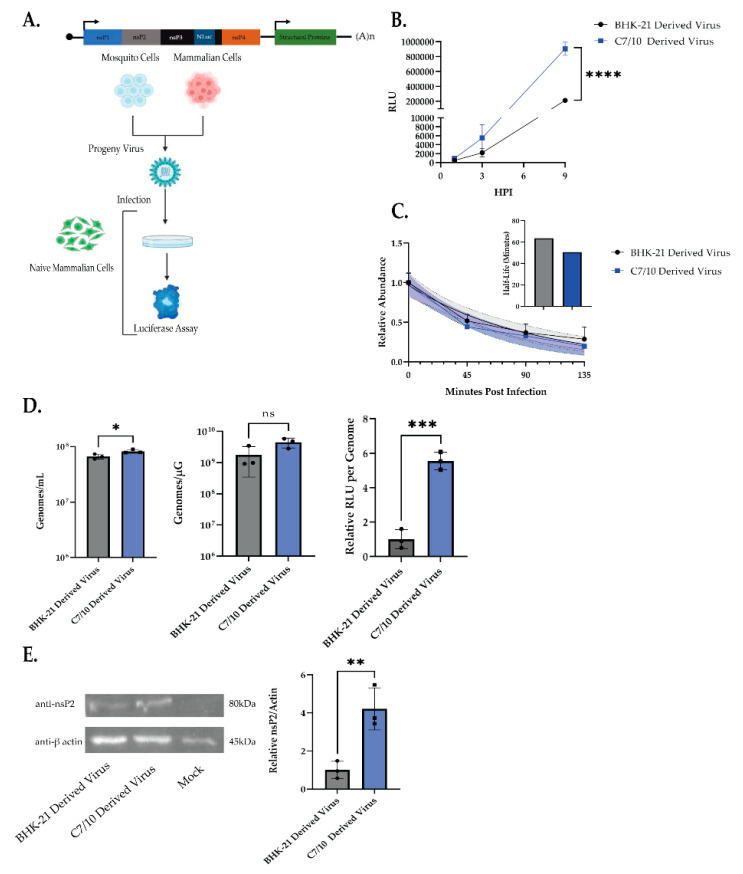
Mosquito-derived virus has increased translation compared to mammalian-derived virus. (**A**) Schematic representation of experiment performed. (**B**) Luciferase experiment performed in HEK-293 cells using Sindbis virus (SINV) carrying a translationally fused nanoluciferase (nLuc) gene in the 5′ open reading frame (ORF1). A total of 10^8^ genomes each of progeny virus derived from BHK-21 and C7/10 cells were used to synchronously infect naïve HEK-293 cells. Cell lysates were collected at indicated times postinfection, and luciferase activity (RLU) was used as a proxy for viral replication. Error bars represent SEM of biological replicates (*n* = 3) Two-way ANOVA using Sidak’s multiple comparisons: time—*p* < 0.0001, BHK/C7-derived virus—*p* < 0.0001, time × BHK/C7-derived virus—*p* < 0.0001. **** *p* < 0.0001. (**C**) Relative abundance of incoming viral RNA in HEK-293 cells over 135 min postinfection was determined by qRT-PCR analysis as described in Materials and Methods. Nonlinear, exponential regression analyses were performed to determine the viral RNA decay profile (represented by solid line). Dashed lines represent the 95% CI of the nonlinear regression. Error bars represent standard error of mean (SEM) of biological replicates (*n* = 3). Two-way ANOVA with Sidak’s multiple comparisons: time—*p* < 0.0001, BHK/C7-derived virus—ns, time × BHK/C7-derived virus—ns. Viral RNA half-life estimated from nonlinear regression (inset). (**D**) Supernatant, total cellular RNA, and cell lysates harvested 9 h postinfection in HEK-293 cells synchronously infected with 10^8^ particles of either BHK-21-derived or C7/10-derived virus was used for qRT-PCR to determine total genome copies per milliliter of supernatant, total genome copies per microgram of cellular RNA, and the ratio of luciferase signal to genomes per microgram present in the same cells. Error bars represent SEM of biological replicates (*n* = 3); statistics determined using Student’s *t*-test. * *p* = 0.045, *** *p* = 0.0005. (**E**) HEK-293 cells were synchronously infected with 10^8^ genomes of either BHK-21- or C7/10-derived virus. Cells were harvested at 9 hpi and used for Western blotting. Band intensity was determined using Image Studio Lite and shown as a ratio of nsP2:β-actin standardized to cells infected with BHK-21-derived virus. Student’s *t*-test: *p* ** = 0.0096.

To examine early molecular events in infection that may contribute to the host-derivation-dependent differences in virus infection, we utilized SINV with a nanoluciferase reporter fused in frame with nsP3 (SINV-nsP3-NLuc), a derivative of SINV p389 [23]. This allows NanoLuc Luciferase activity to be used to measure translation from genomic RNA and has been used as a reporter for replication levels [25,26]. BHK-21- or C7/10-derived SINV-nsP3-NLuc was used to infect HEK-293 cells using 10^8^ particles per well, and luciferase was measured as a surrogate for viral replication (Figure 2A). Following the same trend as observed in the growth curve, the C7/10-derived virus had significantly increased RLU counts compared to the BHK-21-derived virus. Two-way ANOVA with Sidak’s multiple comparisons: time—*p* < 0.0001, BHK/C7-derived virus—*p* < 0.0001, time × BHK/C7-derived virus—*p* < 0.0001 (Figure 2B).

To determine whether the difference in luciferase signal was due to decreased stability of BHK-derived genomic RNA, increased production of C7/10-derived genomic RNA, or enhanced translation of genomic RNA, we examined both incoming genomic vRNA stability and genomic vRNA levels in the cell. To address RNA stability, C7/10- and BHK-21-derived SINV-nsP3-NLuc were used to infected HEK-293 cells in the presence of 4-thiouridine. In the cell, 4SU is converted to 4S-UTP before being incorporated into newly synthesized RNA; thus, any newly synthesized RNA from this point forward would be 4SU-labeled. SINV derived from BHK-21 or C7/10 cells grown in the absence of 4SU, and therefore unlabeled, was then used to synchronously infect the 4SU-treated HEK-293 cells at an MOI of 10 PFU/cell. Following absorption at 4 °C, the cells were washed with 1× PBS three times to remove any unbound virus. Prewarmed (37 °C) media containing 4SU were then added to the cells to initiate viral internalization and infection. Under these conditions, newly synthesized viral and cellular RNA incorporates 4S-UTP, leaving the incoming viral RNA and host RNAs synthesized prior to the addition of 4SU as the only unlabeled RNAs in the cell. All 4SU-labeled RNA was separated from the total cellular RNA pool by biotinylation and streptavidin precipitation. Levels of unlabeled RNA were then measured at each time point as a ratio of vRNA to human 18S using the ΔΔCT method relative to that present at 0 min postinternalization, which represents the initial amount of viral genome delivered. RNA half-life was determined using the nonlinear, exponential decay model (Figure 2C). There was no observed significant difference in the stability of the incoming genomic RNA from either C7/10-derived or BHK-21-derived virus (Figure 2C).

Having observed no difference in the stability of the incoming genomic RNA, we examined production of genomic vRNA in the cell and the amount of vRNA in the supernatant to determine if the increased translation (Figure 2B) was a result of increased replication. To examine this, HEK-293 cells were synchronously infected with 10^8^ particles per well of SINV-nsP3-Nluc, and the cells and supernatant were harvested at 9 h postinfection. Total cellular RNA was extracted and total viral genomic RNA was quantified using qRT-PCR with nsP1 specific primers and a linearized, full-length, plasmid of SINV-nsP3-NLuc to generate a standard curve. Viral genomes were calculated as copies per milliliter of supernatant or per microgram of total cellular RNA (Figure 2D). We found a small but significant difference in the amount of genomic RNA present in the supernatant, but no significant difference in the amount of viral RNA present in the cell. When examining the translation per genomic RNA using the luciferase reporter, we observed a nearly 5.5-fold increase in RLU per genome in the cell (Figure 2D, far right); thus, viral RNA synthesis does not appear to be the cause of the increased luciferase signal (Figure 2D). To further examine differences in the translational efficiency of the viruses, HEK-293 cells were synchronously infected with 10^8^ particles per well, and at 9 h postinfection the cells were harvested and lysed for Western blotting. At 9 h postinfection, there was significantly more nsP2 being expressed in the cells infected with C7/10-derived virus as compared to the cells infected with BHK-21-derived virus (Figure 2E). Quantification of the nsP2 band compared to β-actin showed an approximately 4-fold increase in nsP2 production for the C7/10-derived virus in HEK-293 cells (Figure 2E).

### 3.3. vRNA Is a Determinant of Host Cell Derivation-Dependent Differences in Viral Infection

**Figure 3:**
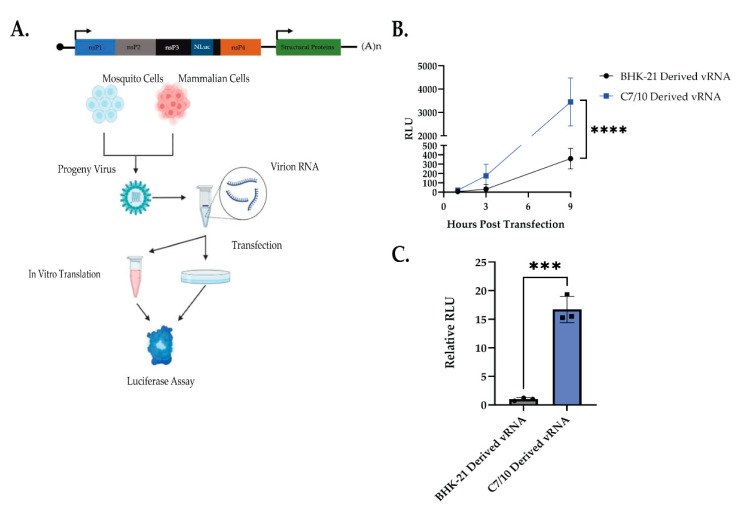
Purified viral RNA phenocopies encapsidated virions. (**A**) Schematic representation of experiment performed. (**B**) A total of 10^8^ copies, determined by qRT-PCR, each of viral RNA extracted from BHK-21- and C7/10-derived viruses were transfected into naïve HEK-293 cells. Cell lysates were harvested at the indicated times and luciferase activity (RLU) was used as a proxy for viral replication. Error bars represent SEM of biological replicates (*n* = 3) Two-way ANOVA using Sidak’s multiple comparisons: Time—*p* < 0.0001, BHK/C7-derived vRNA—*p* = 0.005, time × BHK/C7-derived vRNA—*p* = 0.0004. **** *p* < 0.0001. (**C**) A total of 10^8^ copies each of viral RNA extracted from BHK-21- and C7/10-derived viruses were used for in vitro translation using Rabbit Reticulocyte Lysate (Promega). RLU was standardized to BHK-21-derived vRNA. Error bars represent SEM of biological replicates (*n* = 3). Student’s *t*-test: *p* *** = 0.0003.

As previously mentioned, there are known molecular characteristics acquired by alphaviruses in the mosquito cell that enhance its ability to infect mammalian cells [3,4,5,6]. Thus, to differentiate between the known adaptations and possible vRNA changes, we wished to examine if the vRNA itself was enough to elicit the same phenotype. To determine if the differences observed between the mosquito and mammalian-derived viruses was due to changes in the vRNA alone, we performed transfection experiments of purified vRNA from BHK-21- and C7/10-derived SINV-nsP3-Nluc. SINV-nsP3-Nluc was grown in either BHK-21 or C7/10 cells for 24 or 48 h, respectively (Figure 3A). Virus particles were purified from growth medium as described in Materials and Methods. HEK-293 cells were transfected with 10^8^ viral genome copies of purified vRNA (determined by qRT-PCR) per sample (Figure 3A). Purified vRNA extracted from the C7/10 cell-derived virus and transfected into HEK cells had significantly increased luciferase activity compared to vRNA purified from the BHK-21-derived virus (Figure 3B, two-way ANOVA with Sidak’s multiple comparisons: time—*p* < 0.0001, BHK/C7-derived vRNA—*p* = 0.005, time × BHK/C7-derived vRNA—*p* = 0.0004). These results indicate that the viral genomic RNA is a determinant of enhanced virus infection that was previously observed (Figure 1 and Figure 2), and suggests that there is a host-dependent difference in viral genomic RNA produced.

To confirm that the increased translation is due to differences in the genomic RNA, we performed an in vitro translation assay using purified viral genomic RNA as described in Materials and Methods. Briefly, genomic vRNA was purified and quantified as previously described, and 10^8^ genomes were added to the Rabbit Reticulocyte Lysate per the manufacturer’s instructions. Following a 90 min incubation at 30 °C, luciferase was measured. We found significantly more luciferase activity from the C7/10-derived vRNA template as compared to BHK-21-derived vRNA (Figure 3C).

### 3.4. RNA Modification Profile Is Significantly Different between Mosquito and Mammalian-Derived Virus

**Figure 4:**
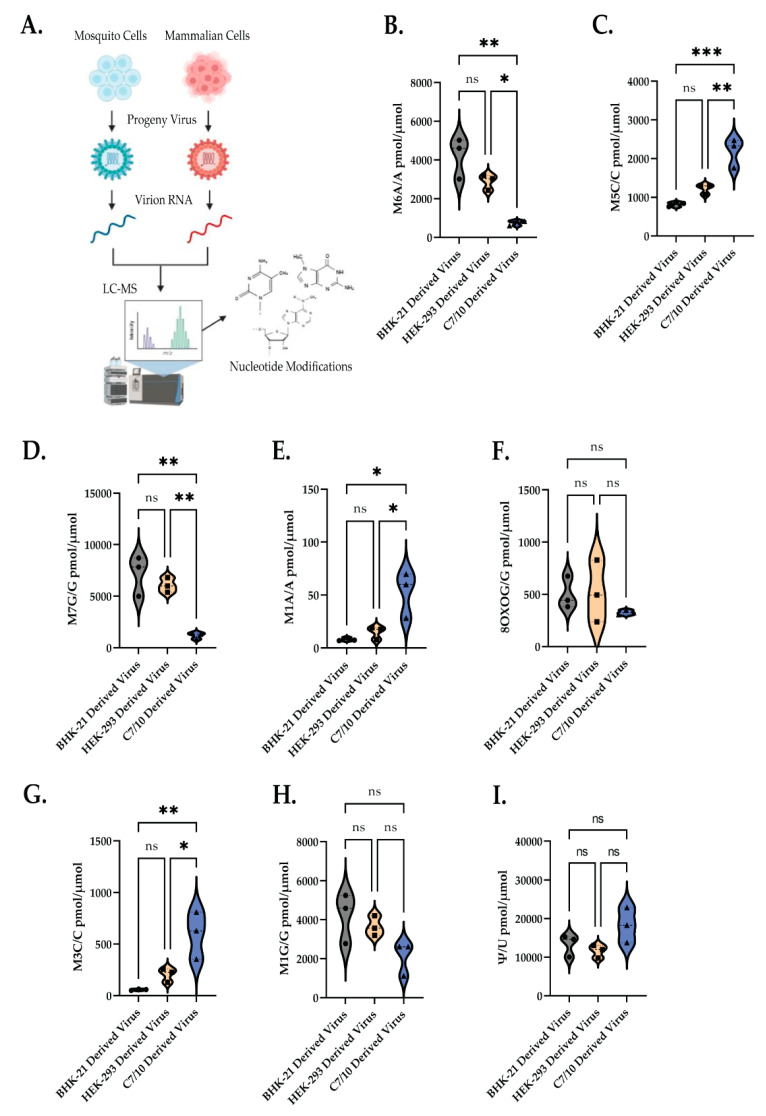
LC/MS analysis reveals species specific modifications acquired by SINV. (**A**) Schematic of liquid chromatography in tandem with mass spectrometry experiment. (**B**) Normalized 6-methyl adenosine (m6A) content of RNA isolated from BHK-21-, HEK-293-, and C7/10-derived virus represented as a ratio of total unmodified adenine content. (*n* = 3) Tukey’s multiple comparison test: *p* * = 0.0176, *p* ** = 0.0016. ns = no significance (**C**) Normalized 5-methyl cytosine (m5C) content of RNA isolated from BHK-21-, HEK-293-, and C7/10-derived virus represented as a ratio of total unmodified cytosine content. (*n* = 3) Tukey’s multiple comparison test: *p* ** = 0.0055, *p* *** = 0.0009. (**D**) Normalized 7-methyl guanine (m7G) content of RNA isolated from BHK-21-, HEK-293-, and C7/10-derived virus represented as a ratio of total unmodified guanine content. (*n* = 3) Tukey’s multiple comparison test: BHK-21 ** *p* = 0.0022, HEK-293 *p* ** = 0.0062. (**E**) Normalized 1-methyl adenosine (m1A) content of RNA isolated from BHK-21-, HEK-293-, and C7/10-derived virus represented as a ratio of total unmodified adenine content. (*n* = 3) Tukey’s multiple comparison test: BHK-21 *p* * = 0.0136, HEK-293 *p* * = 0.0281. (**F**) Normalized 8-oxoguanine (8oxoG) content of RNA isolated from BHK-21-, HEK-293-, and C7/10-derived virus represented as a ratio of total unmodified guanine content. (*n* = 3) Tukey’s multiple comparison test. (**G**) Normalized 3-methyl cytosine (m3C) content of RNA isolated from BHK-21-, HEK-293-, and C7/10-derived virus represented as a ratio of total unmodified cytosine content. (*n* = 3) Tukey’s multiple comparison test *p* * = 0.0309, *p* ** = 0.0074. (**H**) Normalized 1-methyl guanine (m1G) content of RNA isolated from BHK-21-, HEK-293-, and C7/10-derived virus represented as a ratio of total unmodified guanine content. (*n* = 3) Tukey’s multiple comparison test. (**I**) Normalized pseudouridine (Ψ) content of RNA isolated from BHK-21-, HEK-293-, and C7/10-derived virus represented as a ratio of total unmodified uridine content. (*n* = 3) Tukey’s multiple comparison test.

Given that the purified vRNA of the mosquito-derived virus is at least partially responsible for the increased replication in HEK-293 cells, we hypothesized that this may involve host-specific modifications to viral genomic RNA. RNA modifications have been shown to affect a wide range of biological and cellular processes, including—but not limited to—translation of mRNA, structure of RNA, and RNA stability [7,8,9,10,11,12]. Recent work in the field has shown that RNA modifications play an important role in outcome of infection of RNA viruses [14,15]. However, little is known about the RNA modification profile of alphaviruses, or if the RNA modification profile differs between mammalian- and mosquito-derived viruses. To address this, SINV genomic RNA was extracted from purified virions derived from either C7/10, BHK-21, or HEK-293 cells. The vRNA from the purified virions was then subjected to LC-MS/MS analysis of the modified nucleotides in the total genome (Figure 4A). Interestingly, the two mammalian cells produced viruses with very similar quantities of modified nucleotides in the viral genomes, whereas the mosquito-derived virus’ RNA differed in the quantity of a number of modified nucleotides (Figure 4B–I). There was a low amount of m5C modification in the mammalian-cell-derived vRNA and a significantly larger proportion of m5C modification in the mosquito-cell-derived vRNA (Figure 4C). This observation appears to be in line with previous data, which suggests that m5C modification of SINV vRNA is present and proviral in the mosquito vector [15]. Another interesting observation is the significantly higher proportion of m6A modification in mammalian-cell-derived vRNA (Figure 4B). The large proportion of pseudouridine modifications is also interesting, although not unexpected given that it is generally the most frequently found nucleotide modification (Figure 4I). Another interesting observation is the variance seen between modifications (Figure 4B–I). For example, the amount of m6A in the BHK-21- or HEK-293-derived virus is much more variable than the amount in the C7/10-derived virus (Figure 4B). Using mass spectrometry data, it appears that when a modification is largely present, the amount is variable, but when it is largely absent, it is much less variable. Thus, we conclude that cell type has an influential role on the RNA modification profile of SINV progeny, and these differences in RNA modification profiles play a role in increased translation of mosquito-derived vRNA in mammalian cells.

## 4. Discussion

Arboviruses are defined by their capacity to replicate and infect both vertebrate and nonvertebrate hosts in a cyclic infection cycle. Understanding the differences in nonvertebrate- and vertebrate-derived arboviruses and how those differences affect replication is an important step in figuring out how to counteract these viruses. While there is evidence that host origin influences alphavirus infectivity, there is much still to be explored about what happens to the virus after replication in vertebrate or nonvertebrate hosts that primes it for subsequent host infection [2,3,4,5,6]. RNA modifications of viral RNA is a newly emerging field in virology. While modification of vRNA was identified as early as the 1970s, it is only recently that the effect of those modifications on viral replication has begun to be explored [27,28,29]. N6-methyladenosine (m6A) has previously been shown to influence flavivirus replication in mammalian cells, and 5-methylcytosine (m5C) modifications have been shown to influence alphavirus replication in arthropod cells [14,15]. The effects of RNA modifications on the Sindbis virus specifically have not been fully explored, and to that affect, the differences of viral RNA modifications from different host origins have not been examined. This information leads us to ask the following question in this present study: are the differences in replication efficiency of viruses derived from mosquito and mammalian hosts related to the RNA modification state of virion genomic RNA?

Mosquito-derived SINV grows faster and produces more infectious viruses than mammalian-derived SINV in HEK-293 cells (Figure 1). It has been well documented that mosquito-derived arboviruses tend to grow better on mammalian cells than mammalian-derived, and vice versa [24,30]. We have shown that this effect is similar using SINV to infect HEK-293 cells. We observed an early advantage of the mosquito-derived SINV, however, which we decided to explore further (Figure 1C,D).

Luciferase assays showed that mosquito-derived SINV significantly increased translation early in infection (Figure 2B). However, the luciferase assay alone is not enough to conclude whether the early advantage of the mosquito-derived virus is due to translation alone. The increased translation as measured by luciferase assay could be due to increased amounts of genomic vRNA in the cell, which could be due to either the incoming genomic vRNA being more stable, or that the RNA levels are higher. To address these possibilities for the advantage, we looked at both RNA stability having an effect and increased replication of the viral genome (Figure 2C,D). We observed no significant difference in the amount of the genomic viral RNA in the cell infected with the mosquito-derived SINV (~2.5-fold increase) and a small but significant increase in the amount of extracellular vRNA (~1.18-fold increase) (Figure 2D). However, when we measured the amount of luciferase signal as a ratio of genomic vRNA we found a significant increase in the mosquito-derived virus, ~5.5-fold (Figure 2D, far right). While there appears to be a slight increase in the amount of vRNA present in the mosquito-derived virus infection, the relative amount of luciferase produced per genome shows that the mosquito-derived genomic RNA seems to be a better template for translation. Further supporting this observation was the demonstration of an increased amount of nsP2 produced in the cells infected with mosquito-derived virus (Figure 2E), leading us to the conclusion that the differences in RNA modifications are influencing the translational efficiency of the RNA. The results observed in Figure 2 are supported by the ability of purified vRNA alone being sufficient to phenocopy what is seen with intact virus particles, as we see both a significant increase in RLU from purified mosquito-derived genomic RNA transfected into HEK-293 cells, as well as in the in vitro translation assay (Figure 3B,C). Surprisingly, there was no significant difference in the stability of the incoming genomic RNA from mosquito or mammalian-derived viruses, as we know that RNA modifications can affect the stability of some RNAs [10,16,17] (Figure 2C). These results support the hypothesis that acquired RNA modifications from different hosts are also influencing SINV infection, specifically by increasing the translational efficiency of the vRNA, which in turn leads to more infectious viruses being produced. This, along with other known host-dependent molecular modifications of arboviruses, further expand our understanding of the impact of replication background on viral replication and transmission.

The effect of RNA modifications on viral RNA is a newly emerging field with evidence already showing the impact RNA modifications have on the viral life cycle [9,13,14,15,19,20]. There is little known about the effects, let alone the presence, of RNA modifications on alphaviruses specifically or if those modifications are different depending on the host the virus is derived from. We show using liquid chromatography in tandem with mass spectrometry that mosquito-derived SINV and mammalian-derived SINV have significantly different levels of varying RNA modifications. For instance, we see that mosquito-derived SINV s significantly more m5C-modified than the mammalian counterpart, and the opposite is true for m6A modification. This is an interesting find, as previous studies have shown that both m5C and m6A modifications have an impact on arboviruses. More specifically, m5C has been shown to play an important role in the pathogen-blocking ability of *Wolbachia* in insect cells, and m6A modifications have been more extensively studied in the context of mammalian cells [9,13,14,15,19,20]. The difference in the number of m6A modifications in mosquito- and mammalian-derived viruses is consequential for a few reasons: (i) it is in accord with previous data that show that m6A modifications play a role in mammalian cell systems for other arboviruses, and (ii) there is evidence to suggest that m6A modification can help the virus to evade the immune response by escaping RIG-I sensor recognition, which is operative in mammalian cells but not mosquito cells [14,27].

Overall, our results suggest that the RNA modifications acquired by the virus in mosquito cells may play a role in translational efficiency of the viral genome in the mammalian cell. This increased translational efficiency and subsequent increase in infectious virus production is likely influenced by the specific modifications acquired in the mosquito. Further studies will try to identify exactly where these modifications are occurring and look to attribute specific nucleotide modifications to the observed phenotypes. It is likely that each individual type of RNA modification is playing a collective role in the observed phenotypes. While the differences in m5C and m6A modifications are interesting based on the amount of previous research and what is known about these modifications’ effects on arbovirus life cycles, the significant differences in the other observed RNA modifications are just as interesting. While the effects of internal m7G, m3C, m1G, m1A, and Ψ modifications are less well known, the observation of significant differences in some of these modifications implies they also play a role in the viral life cycle, though it is currently difficult to say what role that is. Since each of these modifications affect the base pairing of the modified nucleotide, it is tempting to speculate that they may be found in regions of regulation in which secondary structure is important, and may play important roles in maintaining the appropriate stability of RNA structure under host-specific temperature conditions.

RNA modifications in genomic viral RNA can influence a multitude of things, including—but not limited to—RNA stability, RNA localization, and translational efficiency. Another confounding variable is the difficulty in studying single RNA modifications’ effects, as one modification alone may not be enough to elicit any phenotype. Along those lines, how these RNA modifications interact is not well understood and the possibility of epistatic interactions increases the complexity of analysis and interpretation of biological consequences. Finally, the effect of methylation readers needs to be explored. While there is strong evidence for m6A readers, specifically the YTH family of proteins, having an impact on flavivirus RNA function, there is no reported evidence of the effect readers have on SINV or other alphaviruses [14]. This study opens the door to further studies of the effects that RNA modifications have on arboviruses derived from their arthropod or vertebrate hosts and how those modifications affect RNA function.

## Data Availability

Raw mass spectrometry data have been submitted as Supplemental Material.

## Supplementary Materials

The following supporting information can be downloaded at: https://www.mdpi.com/article/10.3390/v14122606/s1.
